# Improving Ethical Review of Research Involving Incentives for Health Promotion

**DOI:** 10.1371/journal.pmed.1001193

**Published:** 2012-03-27

**Authors:** Alex John London, David A. Borasky, Anant Bhan, for the Ethics Working Group of the HIV Prevention Trials Network

**Affiliations:** 1 Philosophy Department and Center for Ethics and Policy, Carnegie Mellon University, Pittsburgh, Pennsylvania, United States of America; 2 Office of Research Protection, RTI International, Research Triangle Park, North Carolina, United States of America; 3 Researcher, Bioethics and Global Health, Pune, Maharashtra, India

## Abstract

Alex London and colleagues propose new ethical frameworks for evaluating the risks associated with research in which financial or other incentives are used to promote healthy behavior.

**Summary Points**

- Advances in behavioral economics are driving efforts to use material or financial incentives to promote health-related behavior in international development, public health, and clinical medicine.
- Current ethical frameworks for human research assume that material or financial incentives are provided to participants either as compensation for their time and expenses, or as an inducement to participate in research.
- We argue that some common concerns about using incentives to increase participation in research, such as that attractive incentives will undermine participant autonomy, are misplaced when incentives are used to overcome economic obstacles or a lack of effective motivation, and when recipients are incentivized to engage in health-related behaviors or practices with which they are already familiar and which they regard as beneficial or worthwhile.
- We offer additional guidance to research ethics committees aimed at improving the evaluation of research in which incentives are used as an intervention intended to promote healthy behavior.

*Research Integrity Series*

*This is one article in an occasional* PLoS Medicine *series on research integrity that examines issues affecting the ethics of health research worldwide.*

Within international development [1], public health [2], and clinical medicine [3]–[5], there is increasing interest in determining whether cash payments or other economic incentives can be used to influence the choices and behavior of individuals and groups in order to promote desired health goals (Box 1). However, a number of complex issues affect the review and approval by research ethics committees (RECs) of research studying the effectiveness of using financial incentives to promote desired health goals. Current ethical and regulatory frameworks regard the provision of gifts or cash payments to participants in human research as potentially problematic. Specifically, these frameworks imply that such incentives may undermine the autonomy of participant choice, hinder the disclosure of medical information, exacerbate social inequalities, or result in the exploitation or degradation of vulnerable populations [6]–[9]. Typically, these frameworks provide guidance about payment to research participants to reimburse expenses, to compensate for time and effort, to provide insurance coverage, or as an incentive to participate in the research itself. However, the issue of payment as a component of the research *intervention* is relatively new. RECs thus lack explicit guidance about ethical issues surrounding research that evaluates the use of financial incentives as an intervention to promote health.

**Box 1. Context for This Paper**

The authors serve as members of the Ethics Working Group (EWG) of the HIV Prevention Trials Network (HPTN) (http://www.hptn.org/researchethics/ethics.asp). The EWG provides ethics advice for research carried out by the HPTN.

The EWG decided to examine the ethical issues arising from the review of research involving the provision of financial incentives for health for two reasons. First, RECs do not routinely encounter the provision of financial incentives as a health intervention, and there is a lack of guidance about this issue in existing regulations. Second, cash incentives are being used in two ongoing HPTN trials: (1) HPTN-065, a study being conducted in multiple sites in the US that, among other aims, is looking to evaluate the efficacy of financial incentives in promoting expanded HIV testing and linkage to care (http://www.hptn.org/research_studies/hptn065.asp), and (2) HPTN-068, a study being conducted in South Africa to determine whether the provision of cash transfers to young women and their households, conditional on school attendance, reduces young women's risk of acquiring HIV (http://www.hptn.org/research_studies/hptn068.asp).

We argue that when incentives are used to promote healthy behavior there are important cases in which common concerns about the provision of incentives to research participants do not apply, or do not apply with the same force. In these cases, focusing primarily on the size and attractiveness of incentives could lead RECs to make poor decisions. RECs should focus on substantive questions about the likely attitude of recipients to the activity being incentivized, the degree of recipient familiarity with that activity, how risks and benefits are distributed, and whether the incentive raises concerns about fairness or justice. We explore these issues in detail below.

## Types of Payments and Incentives

It is common practice in clinical and public health research to pay participants for expenses incurred as a result of research participation. Payments as reimbursement for expenses like travel costs or parking related to research participation are not generally regarded as ethically problematic. There is, however, a significant literature on how to fairly calculate payments as compensation for time and effort [10]–[12]. Some jurisdictions and institutions also require insurance arrangements so that participants can receive needed care if they are harmed as a result of participation. In each of these cases the goal is to ensure that recipients do not have to incur a financial or material loss as a result of research participation. More controversial, are incentives for research participation: cash payments, prizes, or other material benefits provided for the purpose of encouraging recipients to agree to participate in research (recruitment) or to continue their participation until the study is completed (retention).

Different from all the above are incentives for health promotion: cash payments, gift cards, vouchers, prizes, or other material benefits provided to encourage recipients to utilize or adhere to a health intervention, care plan, or behavior modification activity. The goal is to use the incentive as an intervention intended to produce better health outcomes for individual recipients or better public health outcomes for communities.

When the efficacy of using incentives to promote health benefits is tested in research, some study participants are provided with incentives for health promotion. In this case, RECs may be concerned that an incentive provided with the intention of promoting a specific healthy behavior might also act as an inducement to participate in the study. Our claim is that, even in such cases, ethical judgments should be based on specific factors (discussed below) that determine whether the use of a specific financial incentive creates a moral problem, and how significant that problem is.

## Incentives Sometimes Promote Autonomy

A common concern among RECs is that particularly attractive incentives for research participation, such as large sums of money or difficult-to-obtain medical care, might constitute an undue inducement. If incentives cause recipients to focus myopically on attaining the incentive without attending to other salient aspects of the decision, such as associated risks and burdens, then the recipient's choice would not have the moral significance that comes from the reflective application of their considered values to the full range of relevant information. In this case, incentives might compromise the autonomy of recipients by undermining the integrity of their decision making process.

Whether incentives for research participation compromise the integrity of the recipient's decision making process is largely an empirical question. Recent evidence seems to indicate that such concerns are exaggerated [13]–[18].

Even if we put the empirical evidence aside, however, this concern has the most moral force in cases where incentives are used to influence the choice of a recipient who has not already considered the merits of the available options, and where the course of action being incentivized might pose significant risks to the recipient or involve actions or experiences to which the recipient is averse [19],[20]. Research often uses procedures with which participants are not familiar, such as blinding and random allocation and administration of interventions whose effects may be unknown, and in some cases risks to participants can be significant. In these cases, RECs are rightly sensitive to the potential for incentives for research participation to adversely impact the autonomy of participants.

In many cases, however, incentives for health promotion are used to help recipients bring about a personal change they might already desire, through a means with which they are already familiar. For example, an obese person who is personally committed to losing weight may undertake weight-loss activities but have difficulty adhering to the regimen [21]. The same may be true for smokers or substance abusers trying to quit their addiction [22]. In such cases, cash payments may be used, not to induce the recipient to choose something they would not otherwise choose, but to provide an immediate positive reward for complying with short-term steps necessary to effectuate a longer term goal.

When the purpose of an incentive is to enable recipients to overcome motivational deficiencies in order to effectuate or to maintain a life change to which they are already committed, then, instead of undermining or compromising autonomy, incentives have the potential to be autonomy enhancing [23]. In such cases, the usual prohibition against incentives that are so large that recipients could not refuse them might be both practically self-defeating and of questionable moral value.

Whether particularly large or attractive incentives are required to overcome an agent's inability to achieve a goal that they personally endorse is an empirical question [24]. Programs that rely on such incentives may also be difficult to sustain outside of the trial environment. But the likely effect of an incentive on the autonomy of recipients cannot be determined simply by assessing the incentive's magnitude or attractiveness.

Between explicit aversion and affirmative commitment lie a range of less clear-cut motivational states. Recipients may be motivationally ambivalent, recognizing on some level that an activity is desirable without having an active desire to engage in that activity in practice. Alternatively, recipients may face a motivational conflict, recognizing the importance of several activities or goals that compete for their time and attention. For instance, a patient may desire the long-term health effects of taking a maintenance medication, but may also want to avoid the side effects of continued use. In the context of international development, parents may want to send their child to school but may also want the more immediate social and economic benefits of keeping the child at work.

In cases where recipients have more ambiguous attitudes toward an activity, the ethical assessment of incentives hinges on factors such as the recipient's degree of familiarity with the course of action being incentivized, the distribution and significance of the risks associated with the activity, the nature and distribution of the expected benefits, and whether the program of providing such incentives is equitable and sustainable.

There are many common goals that people might want to accomplish but are unable to maintain the motivation to do so, such as losing weight, breaking addiction, regularly attending school, and practicing safe sex. But it is unlikely that many people share a similar attitude toward research participation. As a result, incentives for research participation may raise ethical concerns that do not arise for incentives to promote healthy behavior. However, knowing that a large incentive might be attainable in a study of incentives for health might induce some people to participate in the research. Whether such an indirect inducement to participate in research would be sufficient to compromise the integrity of the recipient's deliberative process is an empirical question. But the significance of this concern depends on whether the study itself poses significant additional risks to participants and how familiar participants are likely to be with the nature and sources of those risks.

## The Legitimacy of Influencing a Decision

Sometimes REC concerns about undue inducement relate to the legitimacy of interfering with the recipient's deliberation. This concern is not that the incentive causes the recipient to overlook or ignore relevant facts. It is that the desire for the incentive is so strong that it causes the recipient to knowingly act in contravention of deeply held values or beliefs. In this case, the decision reflects the influence of an outside party more than the authentic values of the recipient.

To constitute *wrongful* interference, incentives must be used to prompt recipients to do something that is clearly not in their best interest or to act in a manner that they ought not. For instance, inducing recipients to accept risks that are unreasonably high reflects a lack of respect for the recipients' welfare. It might also be wrong to induce a person to engage in an activity or to accept a risk to which they are averse because doing so constitutes a failure to respect the recipient's view of his or her welfare interests. Finally, an incentive might be viewed as wrongful because it is exploitative, in that it takes unfair advantage of a recipient's vulnerable social or economic position.

Some have argued that by ensuring that research risks are reasonable, prospective REC review significantly diminishes the likelihood that incentives for research participation will constitute wrongful interference [25]. Even with adequate review, however, some worry that large incentives for research participation can induce people to hide information about conditions that would exclude them from participation if disclosed, and that would result in their being exposed to elevated risk [26]. Others worry about the legitimacy of using incentives to overcome participant aversion to research procedures that involve intrusion into or intimate contact with people's bodies or disclosure of sensitive personal information [19],[20].

In many cases, however, incentives for health promotion are offered to help recipients achieve goals that they already desire and from which they directly benefit. In the development context, for example, conditional cash transfers have been used to promote school attendance, improve nutrition, and increase the economic power of women in order to promote gender equality. When recipients stand to benefit directly from such activities, and recipients are unlikely to be averse to the goals they promote, providing financial incentives for health promotion would not constitute wrongful interference. In such cases, even if the prospect of receiving an incentive induces people to enroll in a study of an incentive for health promotion, it is difficult to see how that could constitute wrongful interference.

More difficult questions arise when incentives are used to overcome the motivational indifference, conflict, or aversion of recipients to activities that are most likely to benefit others. In such cases, RECs should consider the importance of the health benefit in question and the likely sources of people's reticence. For example, providing a financial incentive to overcome indifference toward completing the full course of treatment to patients who experience symptomatic relief after only partial completion may be a legitimate means of inhibiting the development of drug resistance in the population. This may be true even if patients are averse to completing the treatment course in order to avoid side effects, as in the case of tuberculosis treatment [27].

## Distribution and Significance of Risks and Potential Benefits

In addition to evaluating whether the provision of an incentive for health promotion is likely to undermine the integrity of the recipient's deliberative process, and whether its use represents a legitimate means of influencing recipient behavior, RECs should pay careful attention to the way that incentives affect the balance of risks and potential benefits in a trial.

Current guidelines state that RECs must ensure that risks to participants are reasonable in light of potential benefits of the research. These benefits may accrue directly to research participants, but this need not be the case [28]. Risks to participants that are not offset by the prospect of direct benefit to participants themselves can still be justified if they have been minimized and are necessary to produce knowledge of sufficient social value [7],[9],[10],[29].

Ethical guidance about the provision of incentives in research holds that financial payments should not be treated as a benefit of research participation that can offset risks to participants. One concern is that allowing incentives for research participation to offset risks that participants may encounter within a study would create a mechanism by which almost any risk or burden could be permitted. This would not only pose extra danger to participants, it would also potentially undermine the integrity of the research enterprise by practically eliminating the only mechanism by which RECs can address the social value of research as a knowledge-generating activity [29]. Research whose social value is insufficient to redeem the risks that it poses to participants could nevertheless be approved by providing sufficiently high payments to research participants. At the extreme, this could result in trials being completed that have little direct value to participants and little or no social value either. Even if participants would stand to profit from receiving the incentive, conducting trials that lack social value undermines the social mission of the research enterprise.

When research examines incentives for health promotion, the incentives are a core component of the study intervention. It would be difficult, therefore, to justify the prohibition against treating them as a benefit during REC review. In the context of international development, for example, incentives such as cash transfers or the provision of food coupons are used to overcome economic deprivations that prevent impoverished individuals from pursuing a development-related activity, such as traveling to a health care center to receive childhood vaccinations, remaining in school, or eating an enriched diet. Receiving the cash or the food in such cases appears to be a direct benefit of the program.

Nevertheless, RECs should be careful when weighing the *significance* of the monetary or material benefit conferred from receiving an incentive for health promotion, especially in cases when the incentive is used to overcome recipient aversion to risks associated with the activity being incentivized. In particular, if the benefits of achieving the incentivized goal for the individual, or for the larger community, are not sufficient to justify the risks associated with a trial, then the provision of material incentives to promote those goals should not be treated as a sufficiently significant additional benefit to alter the unfavorable risk–benefit ratio of the trial as a whole.

RECs should also consider whether providing material incentives will negatively affect the risk–benefit profile of an activity. For example, if incentives can be easily linked to a treatment program or research protocol, they might reveal sensitive information that increases the risk that recipients will suffer adverse social consequences. Similarly, in resource-poor settings the knowledge that someone is the recipient of a cash transfer may make that person a target for robbery or assault. The use of incentives in programs to enhance gender equality may also place female recipients at elevated risk of violence if male community members seek to reinforce operant social norms with violence. Some claim that inducing people to engage in a health-promoting activity via the motive of profit might “crowd out” whatever intrinsic motivation the recipient might have for engaging in that activity [30]–[32]. Similarly, financial incentives might promote expectations of financial dole-outs for all research projects and/or health interventions, irrespective of the scientific merit of using incentives in those cases. Another concern is that financial incentives invite strategic behavior in which some individuals attempt to game the system.

RECs should evaluate the plausibility of predictions about these risks in light of the most current empirical findings, and research should seek to evaluate the degree to which such risks materialize in actual practice.

## Fairness and Social Justice

Several issues discussed above are tightly connected to considerations of fairness and justice. For example, the risk–benefit profile of a trial is an important moral concern in part because the welfare of participants is itself of fundamental moral import. But it is also important for ensuring that the research enterprise reflects fair terms of social cooperation, and merits the trust and support of the myriad stakeholders who contribute to its ability to serve the common good [33]. It should not be the case, for example, that risks are primarily borne by the disadvantaged or socially marginalized while the benefits of innovation accrue to those who are already comparatively advantaged [6].

Although the prospect that financial incentives could be used to improve health outcomes is an attractive hypothesis in a variety of domains, careful consideration must be given to the sustainability of such interventions. Because communities and health systems may differ in what kind of health interventions they can deploy on a sustainable basis, application of the same moral standards across communities may yield different assessments of the ethics of conducting the same trial. In particular, investigating the merits of an intervention involving financial incentives may represent a poor use of scarce resources in communities that could not deploy the intervention on a sufficient level to achieve the relevant health objective.

A lack of relevance to the health needs of the host community may also affect the risk–benefit assessment of a trial. In part, as the social value of the trial decreases, it becomes more difficult to justify risks to participants that are not offset by the prospect of direct benefit. There is also evidence that introducing and then removing a material incentive for an activity can crowd out existing motivation for engaging in that activity [34],[35]. For this reason, even when the provision of financial incentives is sustainable in a population, careful consideration should be given to the plan for terminating research and rolling out the associated intervention.

When research is relevant to the health needs of the host community, participating in and supporting the research enterprise can be seen as a way of contributing to an important public good [33]. When significant disparities exist between research sponsors and host communities, these features of responsiveness create the foundation for a collaborative partnership in which all stakeholders can be seen as the moral equal of the others. Alternatively, if recipients view incentives as a mechanism for constraining their agency in order to advance the interests of those offering the incentive, then incentives can inhibit compliance and crowd out intrinsic motivation [32]. When clearly linked to health or development goals that can be seen as empowering recipients, such incentives are more likely to encourage crowding in of pro-health behaviors.

Finally, monetary or material incentives can themselves have a direct impact on the distribution of opportunities in a community, at least to the extent that the incentive increases the basket of resources available to recipients. In some cases, this can help to mitigate larger social inequalities that may disadvantage poor or marginalized populations and reduce economic pressures that lead impoverished individuals to engage in high-risk behaviors (such as the reliance of adolescent girls on older men who promise to provide them with economic support in return for sexual favors, which makes them susceptible to exploitation and HIV infection).

Nevertheless, when incentives for health promotion are evaluated in trial designs that involve the use of a non-incentive control arm, care must be taken to ensure that the research does not generate or exacerbate objectionable inequalities in the host community.

## Conclusion

Whether paying people to engage in pro-health behaviors represents an effective, sustainable, and cost-effective tool for promoting individual and public health is an important research question. When incentives are used to encourage utilization of, or compliance with, established means of producing individual or public health benefits and when it is likely that recipients are already favorably disposed to these goals, then traditional concerns about the provision of incentives in research may be misplaced, and even misguided. When trials are more complex, involving multiple interventions or interventions that are unfamiliar or investigational, RECs need to pay careful attention to the considerations outlined above (Box 2).

**Box 2. Policy Recommendations for REC Review of Research Involving Incentives for Health Promotion**

1. Before considering the amount or potential attractiveness of an incentive, RECs should consider the attitude of recipients to the activity being incentivized and the degree of recipient familiarity with that activity.
2. We propose that concerns around the potential for incentives to undermine recipient autonomy are misplaced when incentives are used to overcome economic obstacles or a lack of effective motivation, and when recipients are incentivized to engage in health-related behaviors or practices with which they are already familiar and which they regard as beneficial or worthwhile.
3. It may be appropriate to treat the receipt of a financial or material incentive as a benefit when reviewing research in which the incentive is itself a component of the health intervention. However, if the benefits of achieving the incentivized goal for the individual, or for the larger community, are not sufficient to justify the risks associated with a trial, then receiving the incentive should not be treated as a sufficiently significant additional benefit to alter the unfavorable risk–benefit ratio of the trial as a whole.
4. RECs should require researchers to provide an evidence-based rationale for predicting that the provision of an incentive will encourage the intended health behavior and not adversely affect the willingness of participants or community members to engage in that behavior. This rationale should also assess the likely effects on participants and communities of the withdrawal of the incentive during or at the conclusion of the study. Where possible, studies should gather the data necessary to evaluate the accuracy of this rationale.
5. RECs should ensure, as far as possible, that the use of incentives to promote healthy behavior could be sustained in the context where research is conducted and would not represent an unreasonable use of scarce health resources.

## Abbreviations

EWG: Ethics Working Group
HPTN: HIV Prevention Trials Network
REC: research ethics committee

## References

[1] Fiszbein A, Schady N (2009). Conditional cash transfers: reducing present and future poverty.

[2] Lagarde M, Haines A, Palmer N (2007). Conditional cash transfers for improving uptake of health interventions in low- and middle-income countries: a systematic review.. JAMA.

[3] Lussier JP, Heil SH, Mongeon JA, Badger GJ, Higgins ST (2006). A meta-analysis of voucher-based reinforcement therapy for substance use disorders.. Addiction.

[4] Giuffrida A, Torgerson DJ (1997). Should we pay the patient? Review of financial incentives to enhance patient compliance.. BMJ.

[5] Paul-Ebhohimhen V, Avenell A (2008). Systematic review of the use of financial incentives in treatments for obesity and overweight.. Obes Rev.

[6] National Commission for the Protection of Human Subjects of Biomedical and Behavioral Research (1979). The Belmont report.

[7] 59^th^ WMA General Assembly (2008). WMA Declaration of Helsinki: ethical principles for medical research involving human subjects.

[8] Council for International Organizations of Medical Sciences, World Health Organization (2002). International ethical guidelines for biomedical research involving human subjects.

[9] US Department of Health and Human Services, National Institutes of Health, Office for Human Research Protections (2005). The common rule, Title 45 (public welfare), Code of Federal Regulations, Part 46 (protection of human subjects).. http://ohsr.od.nih.gov/guidelines/45cfr46.html.

[10] Ackerman T (1989). An ethical framework for the practice of paying research subjects.. IRB.

[11] Dickert N, Grady C (1999). What's the price of a research subject? Approaches to payment for research participation.. N Engl J Med.

[12] Dickert N, Emanuel E, Grady C (2002). Paying research subjects: an analysis of current policies.. Ann Intern Med.

[13] Halpern SD, Raz A, Kohn R, Rey M, Asch DA (2010). Regulated payments for living kidney donation: an empirical assessment of the ethical concerns.. Ann Intern Med.

[14] Cryder CE, John London A, Volpp KG, Loewenstein G (2010). Informative inducement: study payment as a signal of risk.. Soc Sci Med.

[15] Singer E, Couper MP (2008). Do incentives exert undue influence on survey participation? Experimental evidence.. J Empir Res Hum Res Ethics.

[16] Anderson EE, DuBois JM (2007). The need for evidence-based research ethics: a review of the substance abuse literature.. Drug Alcohol Depend.

[17] Bentley JP, Thacker PG (2004). The influence of risk and monetary payment on the research participation decision making process.. J Med Ethics.

[18] Halpern SD, Karlawish JH, Casarett D, Berlin JA, Asch DA (2004). Empirical assessment of whether moderate payments are undue or unjust inducements for participation in clinical trials.. Arch Intern Med.

[19] Grant RW, Sugarman J (2004). Ethics in human subjects research: do incentives matter?. J Med Philos.

[20] London AJ (2005). Undue inducements and reasonable risks: will the dismal science lead to dismal research ethics?. Am J Bioeth.

[21] Volpp KG, John LK, Troxel AB, Norton L, Fassbender J (2008). Financial incentive-based approaches for weight loss: a randomized trial.. JAMA.

[22] Volpp KG, Troxel AB, Pauly MV, Glick HA, Puig A (2009). A randomized, controlled trial of financial incentives for smoking cessation.. N Engl J Med.

[23] Marteau TM, Ashcroft RE, Oliver A (2009). Using financial incentives to achieve healthy behavior.. BMJ.

[24] Filmer D, Schady N (2009). Are there diminishing returns to transfer size in conditional cash transfers? Policy Research Working Paper Series 4999.

[25] Emanuel EJ (2005). Undue inducement: nonsense on stilts?. Am J Bioeth.

[26] Macklin R (1981). “Due” and “undue” inducements: on paying money to research subjects.. IRB.

[27] Awofeso N (2008). Anti-tuberculosis medication side-effects constitute major factor for poor adherence to tuberculosis treatment.. Bull World Health Organ.

[28] Weijer C (1999). Thinking clearly about research risk: implications of the work of Benjamin Freedman.. IRB.

[29] London AJ, Kimmelman J, Emborg ME (2010). Research ethics. Beyond access vs. protection in trials of innovative therapies.. Science.

[30] Deci EL, Koestner R, Ryan RM (1999). A meta-analytic review of experiments examining the effects of extrinsic rewards on intrinsic motivation.. Psychol Bull.

[31] Frey BS, Jegen R (2001). Motivation crowding theory.. J Econ Surv.

[32] Bowles S (2008). Policies designed for self-interested citizens may undermine “the moral sentiments”: evidence from economic experiments.. Science.

[33] London AJ (2005). Justice and the human development approach to international research.. Hastings Cent Rep.

[34] Murayama K, Matsumoto M, Izuma K, Matsumoto K (2010). Neural basis of the undermining effect of monetary reward on intrinsic motivation.. Proc Natl Acad Sci U S A.

[35] Camerer CF (2010). Removing financial incentives demotivates the brain.. Proc Natl Acad Sci U S A.

